# The E545K mutation of PIK3CA promotes gallbladder carcinoma progression through enhanced binding to EGFR

**DOI:** 10.1186/s13046-016-0370-7

**Published:** 2016-06-18

**Authors:** Shuai Zhao, Yang Cao, Shi-bo Liu, Xu-an Wang, Run-fa Bao, Yi-jun Shu, Yun-ping Hu, Yi-jian Zhang, Lin Jiang, Fei Zhang, Hai-bin Liang, Huai-feng Li, Qiang Ma, Yi Xu, Zheng Wang, Yi-chi Zhang, Lei Chen, Jian Zhou, Ying-bin Liu

**Affiliations:** Department of General Surgery and Laboratory of General Surgery, Xinhua Hospital, Affiliated to Shanghai Jiao Tong University, School of Medicine, Shanghai, 200092 People’s Republic of China; Institute of Biliary Tract Diseases Research, Shanghai Jiao Tong University School of Medicine, Shanghai, 200092 People’s Republic of China; Department of General Surgery, Xinhua Hospital Affiliated to Shanghai Jiao Tong University School of Medicine, 1665 Kongjiang Road, Shanghai, 200092 China

**Keywords:** Cancer, PI3K, EGFR, Gallbladder carcinoma

## Abstract

**Background:**

Gallbladder carcinoma (GBC) is the most common malignancy of the bile duct and patients with GBC have extremely poor prognoses. PIK3CA, which encodes the phosphoinositide 3-kinase (PI3K) subunit p110α, is frequently mutated in many cancers, including GBC. The function of the E545K mutation in GBC is not fully understood.

**Methods:**

E545K mutation was determined in human GBC tissues by targeted sequencing. The effects of E545K mutation and PI3K selective inhibitor, A66 on GBC cells were evaluated using Cell Counting Kit-8 (CCK-8) cell Viability and transwell assays. The mechanisms of E545K mutation and A66 were analyzed by western blot and co-immunoprecipitation (Co-IP) assay. Subcutaneous xenograft models in nude mice were employed to evaluate the role of E545K mutation and A66 in GBC progression.

**Results:**

The rate of PIK3CA E545K mutation in GBC patients was 6.15 %. And the survival of GBC patients was correlated with E545K mutation significantly (*P* < 0.05). The E545K mutation promoted proliferation, migration and invasion of GBC cells in vitro and tumor proliferation in vivo. A66 suppressed proliferation of GBC cells in vitro and tumor proliferation in vivo.

**Conclusion:**

The prognoses of patients with E545K mutation were worse than patients without this mutation. The E545K mutation promoted GBC progression through enhanced binding to EGFR and activating downstream akt activity. The PI3K selective inhibitor, A66, suppressed gallbladder carcinoma proliferation.

**Electronic supplementary material:**

The online version of this article (doi:10.1186/s13046-016-0370-7) contains supplementary material, which is available to authorized users.

## Background

Gallbladder carcinoma (GBC) is the most common malignancy of the bile duct and the fifth most common gastrointestinal cancer [1, 2]. Patients with GBC have extremely poor prognoses, and their 5-year survival rate is less than 10 % [3, 4]. There is currently no effective drug for the treatment of patients with GBC, thus, novel effective drugs are urgently needed to improve the prognoses of these patients. Precision-medicine has been deemed increasingly important for clinical treatment [5], and the identification of the mechanisms of the development and progression of GBC is needed to improve the prognoses of patients with GBC.

Phosphoinositide 3-kinases (PI3Ks) are key components of cell signaling pathways that regulate proliferation and apoptosis and play important roles in the proliferation, invasion and metastasis of cancer cells [6, 7]. Stimulated by upstream receptor tyrosine kinases (RTKs) and G protein-coupled receptors (GPCRs), PI3Ks transduce signals into intracellular messages that then activate AKT and other downstream effector pathways [8]. Class Ia PI3Ks have been widely studied and are thought to have the most important effects among all PI3Ks [8]. Class Ia PI3Ks contain a p110 catalytic subunit and a p85 regulatory subunit. Three homologous p110 catalytic isoforms (p110α, p110β and p110δ) are encoded by three different genes: PIK3CA, PIK3CB and PIK3CD. The p110α and p110β subunits are ubiquitously expressed, whereas the p110δ subunit is largely restricted to the immune system in mammals [6]. Upon growth factor stimulation, p110α binds to the phospho-tyrosine residues of receptor protein kinases or adaptor proteins through interactions with p85 and subsequently activates the lipid kinase activity of p110α [9]. Activated p110α converts phosphatidylinositol-4,5-bisphosphate (PIP2) to phosphatidylinositol-3,4,5-triphosphate (PIP3), and PIP3 subsequently activates the downstream AKT signaling pathway as a second messenger [9, 10]. Recent cancer studies have revealed that many components of the PI3Ks, including p110α, are frequently targeted by germline or somatic mutations in a variety of human cancers. These findings and the fact that PI3Ks are highly suited for pharmacologic intervention make the PI3K pathway one of the most attractive targets for therapeutic cancer interventions [11]. Numerous PI3K inhibitors have been tested in clinical trials in recent years, however, inhibitor resistance has been widely observed [12–15]. Most somatic PIK3CA mutations in human cancers occur within two hot spots: E545K and H1047R. The E545K mutation of PIK3CA was first reported in 2005 [16]. Previous studies have identified the E545K mutation of PIK3CA in various carcinomas, including colorectal cancer, glioblastoma, gastric cancer, breast cancer, lung cancer [17], esophageal squamous cell cancer [18], pancreatic cancer [19], intrahepatic cholangiocarcinoma [20] and GBC [21]. Recently, we found that E545K is the only missense mutation of PIK3CA in GBCs based on whole-exome and targeted genesequencing [21]. The function of the E545K mutation in GBC cells is not been fully understood.

In this study, we analyzed the PIK3CA E545K mutation in a cohort of GBC samples and explored the function of the PIK3CA E545K mutation in GBC. Moreover, we tested the effect of a PI3K inhibitor on GBC.

## Results

### Patients with GBC harboring the E545K mutation exhibit worse prognoses

**Fig. 1:**
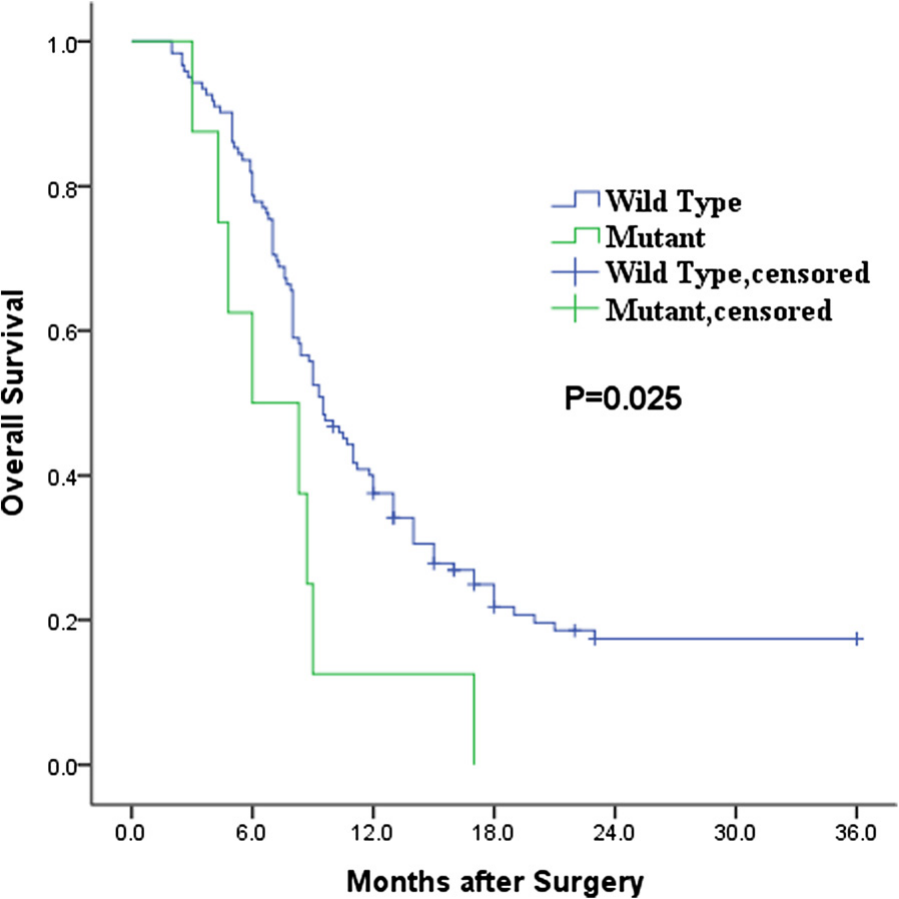
Kaplan–Meier curves for the overall survival of GBC patients according to their wild type (*n* = 122) or mutation (*n* = 8)

In previous work, we found that three of 57 patients with GBC harbored the PIK3CA E545K mutation [21]. To determine the effect of the E545K mutation, 130 patients with GBC received follow-up for 36 months after surgery, and we identified eight patients with the E545K mutation via targeted sequencing of the resected tumors (shown in Additional file 1: Table S1). The clinical data for the patients are provided in Additional file 1: Table S1. We found that the E545K mutation was not significantly associated with age, gender, lymph node metastasis, TNM(tumor, node, metastasis) stage, margin status or jaundice (Table 1). However, this mutation was significantly associated with the patients’ overall survival (Fig. 1). Thus, we sought to determine the function of the E545K mutation in GBC.

**Table 1:** Relationships of the PIK3CA E545K mutation with the clinicopathological characteristics of the GBCs

Parameter	Category	No.of cases	PI3K E545K mutation status		
			No.of positive cases (%)	χ2	*P* value
Age	<60	60	3 (5 %)	0.020	0.888
	≥60	70	5 (7.14 %)		
Gender	Male	61	3 (4.92 %)	0.034	0.853
	Female	69	5 (7.25 %)		
Lymph node metastasis	Negative	86	4 (4.65 %)	0.373	0.541
	Positive	44	4 (9.09 %)		
TNM stage	II	8	0 (0 %)	(a)	0.352
	III	101	6 (5.94 %)		
	IV	21	2 (9.52 %)		
Margin status	R0	71	5 (7.04 %)	0.009	0.924
	R1	59	3 (5.08 %)		
Jaundice	Negative	91	6 (6.59 %)	0.000	1.000
	Positive	39	2 (5.13 %)		

(a) The TNM stage data did not meet the criterion for χ2 analysis, thus, we used a Spearman rank correlation analysis

### PIK3CA is vital for the proliferation, migration, invasion and clone formation of GBC cells in vitro

To investigate the function of PIK3CA in GBC, we knocked down PIK3CA in GBC cell lines. We assessed PIK3CA expression in 6 GBC cell lines and found that the expression quantities in the 6 GBC cell lines were similar (Additional file 1: Figure S1a). The GBC-SD and NOZ cell lines were used in following experiments. Three different siRNAs were designed to knockdown PIK3CA (Additional file 1: Figure S1b, c). siRNA2 (referred to as siRNA below) exhibited the greatest knockdown efficiency and was therefore used in the following experiments.

**Fig. 2:**
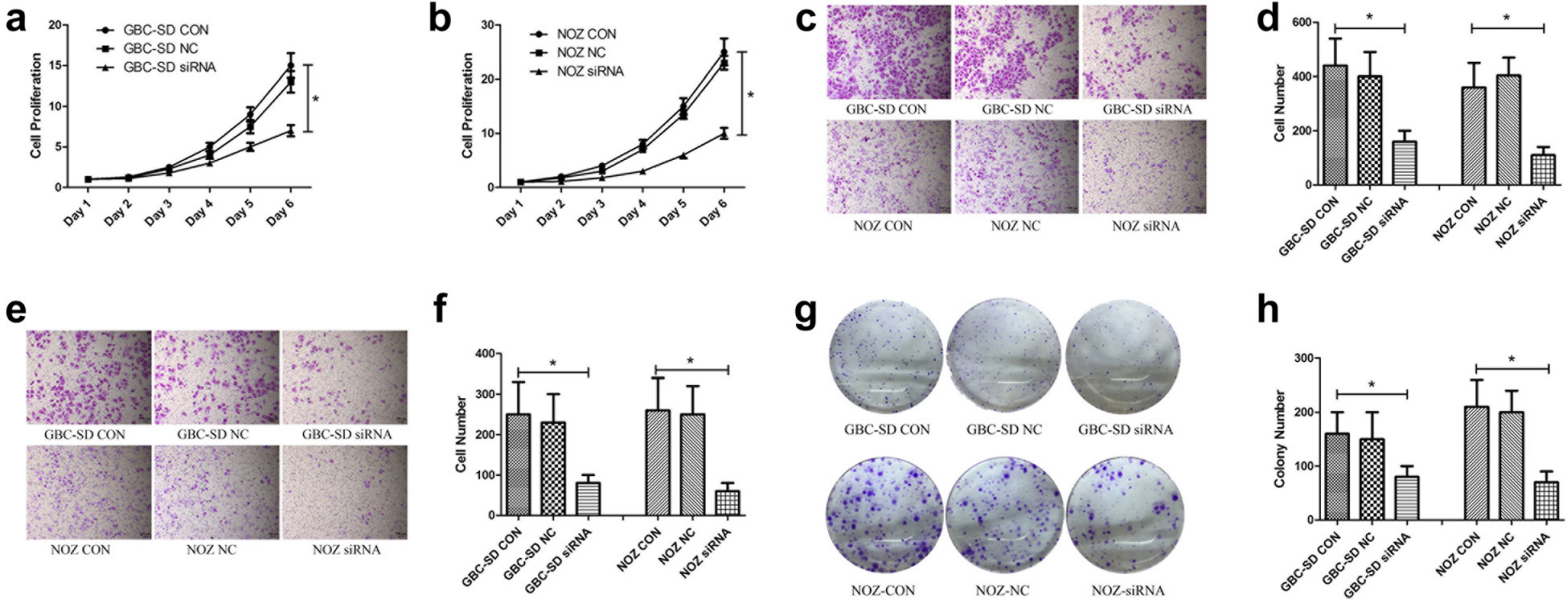
PIK3CA is vital for the proliferation, migration, invasion and clone formation of GBC in vitro. **a**, **b** GBC-SD and NOZ cells treated with CON, NC and siRNA were evaluated via a CCK8 cell viability assay. Cell proliferation was represented as cell quantity. Data are presented as mean ± SD (*n* = 5). **c**, **d** Migration assays of the GBC-SD and NOZ cells treated with CON, NC and siRNA were evaluated with uncoated transwells. Representative stained images of migrated cells and the numbers of migrated cells (mean ± SD, *n* = 5) are shown. **e**, **f** Invasion assays of the GBC-SD and NOZ cells treated with CON, NC and siRNA were evaluated with Matrigel-coated transwells. Representative images of stained invaded cells and the numbers of invaded cells (mean ± SD, *n* = 5) are shown. **g**, **h** GBC-SD and NOZ cells were treated with CON, NC and siRNA and allowed to form colonies. Representative stained images and the colony numbers (mean ± SD, *n* = 3) are shown

After the knockdown of PIK3CA with siRNA, the proliferations of the GBC-SD and NOZ cells were obviously suppressed (Fig. 2a, b). Moreover, the knockdown of PIK3CA significantly suppressed the migration and invasion of GBC-SD and NOZ cells (Fig. 2c-f). We also found that GBC-SD and NOZ clone formation was suppressed following the knockdown of PIK3CA (2 g, 2 h). Together, these findings demonstrated that PIK3CA plays an indispensable role in GBC progression.

### The E545K mutation of PIK3CA promotes GBC progression in vitro

To determine whether the E545K mutation of PIK3CA affects GBC progression, we transfected PIK3CA-WT and PIK3CA-E545K plasmids into GBC-SD and NOZ cells. We conducted reverse transcription polymerase chain reaction (RT-PCR) and Western blot and observed similar PI3K p110α expression levels in PIK3CA-E545K and PIK3CA-WT cells (Additional file 1: Figure S2a-c).

**Fig. 3:**
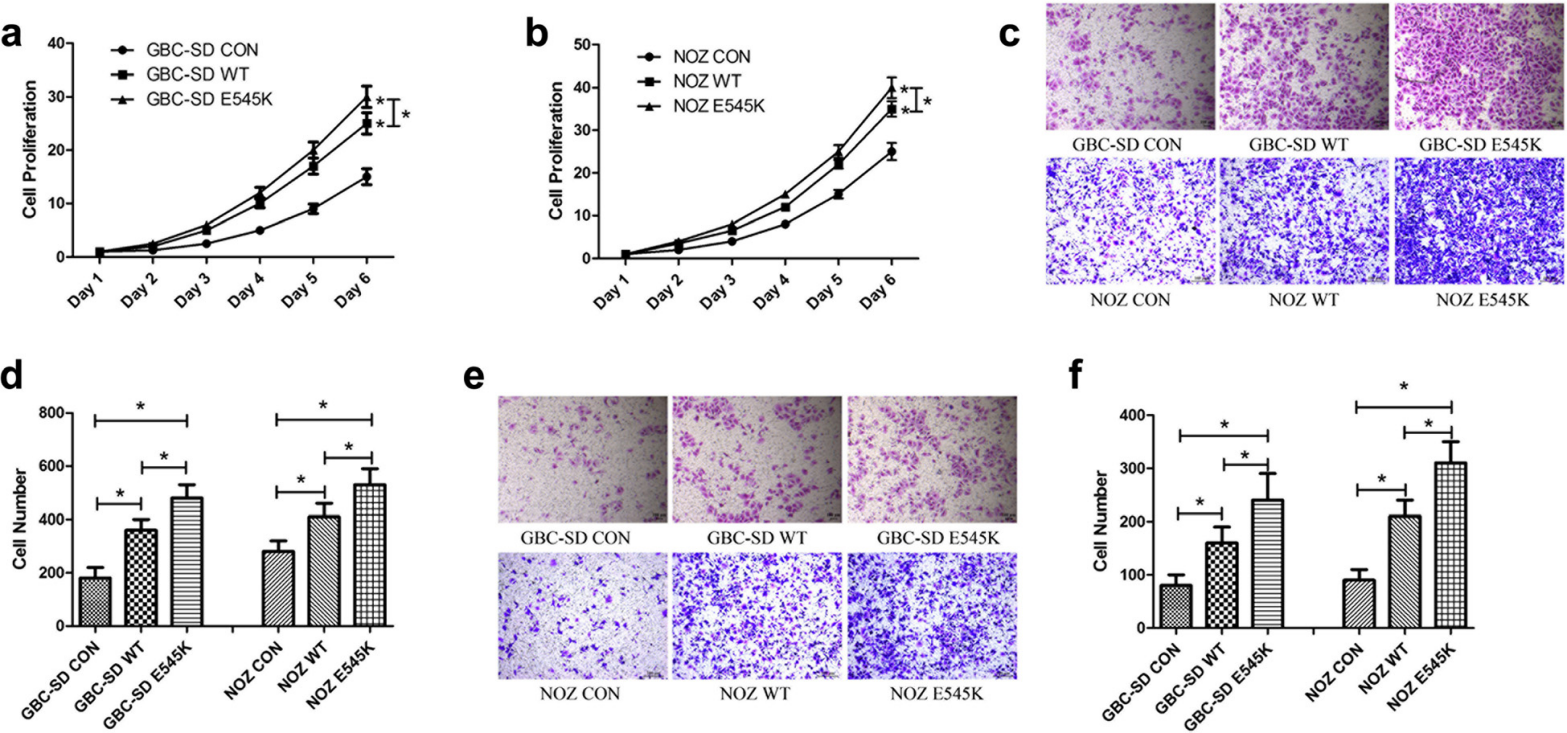
The E545K mutation of PIK3CA promotes GBC progression in vitro. **a**, **b** GBC-SD and NOZ cells treated with CON, WT and E545K were evaluated using CCK8 cell viability assays. Cell proliferation was represented as cell quantity. Data are presented as mean ± SD (*n* = 5). **c**, **d** Migration assays of the GBC-SD and NOZ cells treated with CON, WT and E545K were evaluated with uncoated transwells. Representative images of the stained migrated cells stained pictures and the numbers of migrated cells (mean ± SD, *n* = 5) are shown. **e**, **f** Invasion assays of the GBC-SD and NOZ cells treated with CON, WT and E545K were evaluated with Matrigel-coated transwells. Representative images of stained invaded cells and the numbers of invaded cells (mean ± SD, *n* = 5) are shown

The overexpression of either PIK3CA-WT or PIK3CA-E545K significantly increased cell proliferation, migration and invasion in the GBC-SD and NOZ cells (Fig. 3a-f). Compared with the WT cells, the E545K mutation elicited greater effects in both the GBC-SD and NOZ cells in terms of accelerating cells proliferation (Fig. 3a, b). Moreover, the E545K mutation accelerated cell migration and invasion more strongly in both the GBC-SD and NOZ cells compared with the WT cells (Fig. 3c-f).

### The E545K mutation of PIK3CA increased downstream Akt activity

**Fig. 4:**
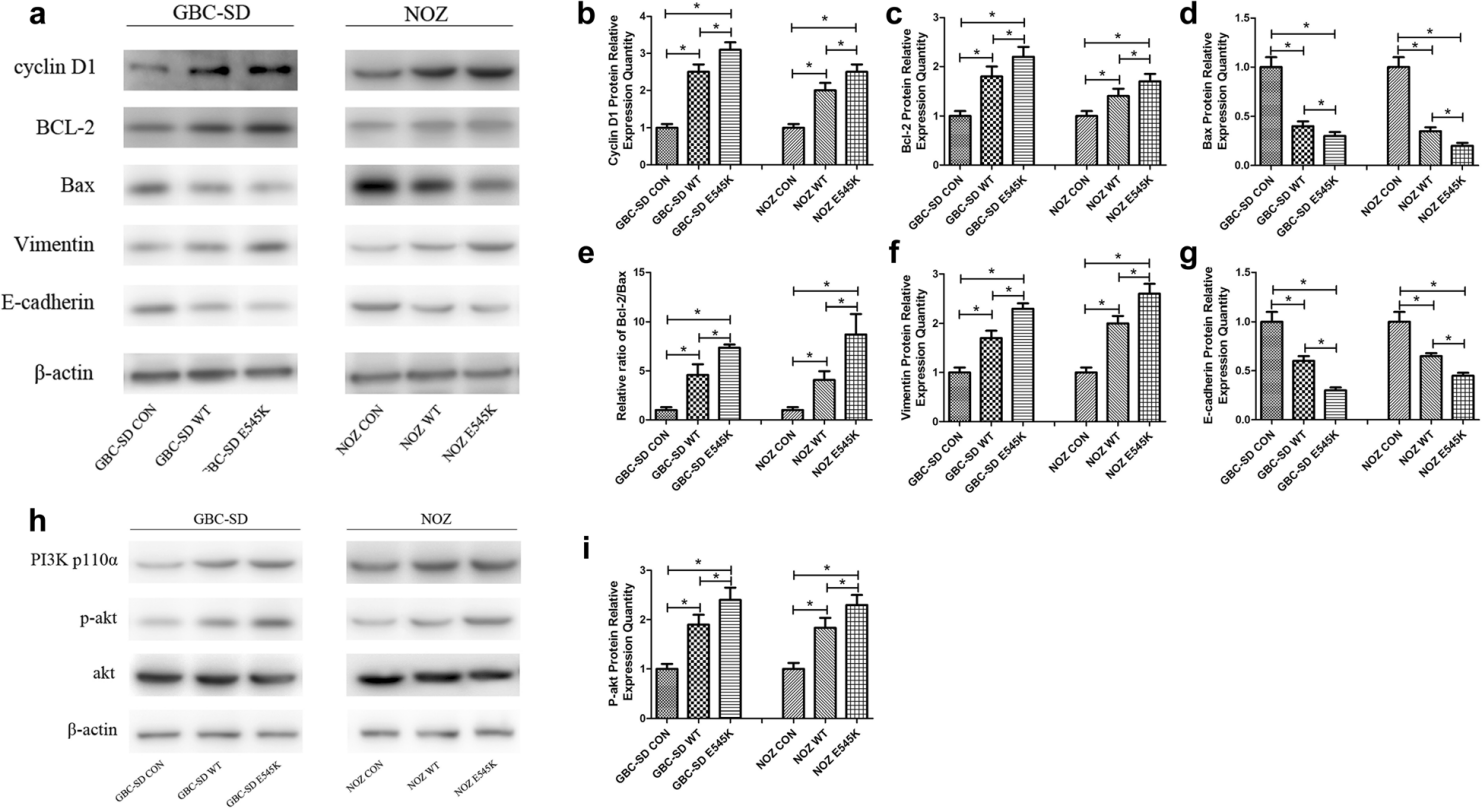
The E545K mutation of PIK3CA increased downstream Akt activity. **a**-**g** The functional proteins associated with the PI3K-akt pathway in cancer progression were examined by Western blot assays. Representative chemiluminescent images and the relative expression quantities of cyclin D1, bcl-2, Bax, vimentin and E-cadherin (mean ± SD, *n* = 3) are shown. **h**, **i** Key proteins in the PI3K-akt pathway were examined by Western blot assays. Representative chemiluminescent images of PI3K p110α, p-akt and akt and the relative expression quantities of p-akt (mean ± SD, *n* = 3) are shown

We first investigated the proteins in the PI3K-akt pathway that were functionally associated with cancer progression. In agreement with the cell proliferation experiments, the E545K mutation increased the expression of cyclin D1 (Fig. 4a, b), which demonstrated that the E545K mutation promoted the cell cycle and thus improved cell proliferation. Furthermore the E545K mutation increased Bcl-2 expression and decreased Bax expression. The Bcl-2/Bax ratio of the E545K mutants was significantly greater than that of the WTs (4a, 4c-4e), suggesting that the E545K mutation suppressed cell apoptosis. We also found that the E545K mutation increased vimentin expression and decreased E-cadherin expression (Fig. 4a, f and g). These results indicate that the E545K mutation improved cell migration and invasion, which is consistent with the observations from the cytological experiments.

Based on the experiments described above, we found that E545K-PIK3CA and WT-PIK3CA cells exhibited abnormal cytological and protein behaviors. These findings suggested that the E545K mutation may enhance the activity of the PI3K-Akt pathway. Therefore, we investigated total-Akt and p-Akt expression. As expected, p-Akt expression in the E545K was higher than WT (Fig. 4h, i), indicating that the E545K mutation improved Akt activity.

### The E545K mutation enhanced the binding of PI3K p110α to epidermal growth factor receptor (EGFR)

After obtaining the above results, we further sought to examine how the E545K mutation increased Akt activity. P110α exerts its effects via recruitment to the membrane via the interaction of the p85 subunit with the tyrosine phosphate motifs receptors that are activated in response to growth factor stimulation and the subsequent activation of RTKs [9, 10]. EGFR is a primary member of the ERBB family, which is a group of extremely important RTKs. To explore the potential mechanism underlying our findings, we hypothesized that the E545K mutation enhances p110α binding to EGFRs and subsequently increases PI3K-Akt pathway activity.

**Fig. 5:**
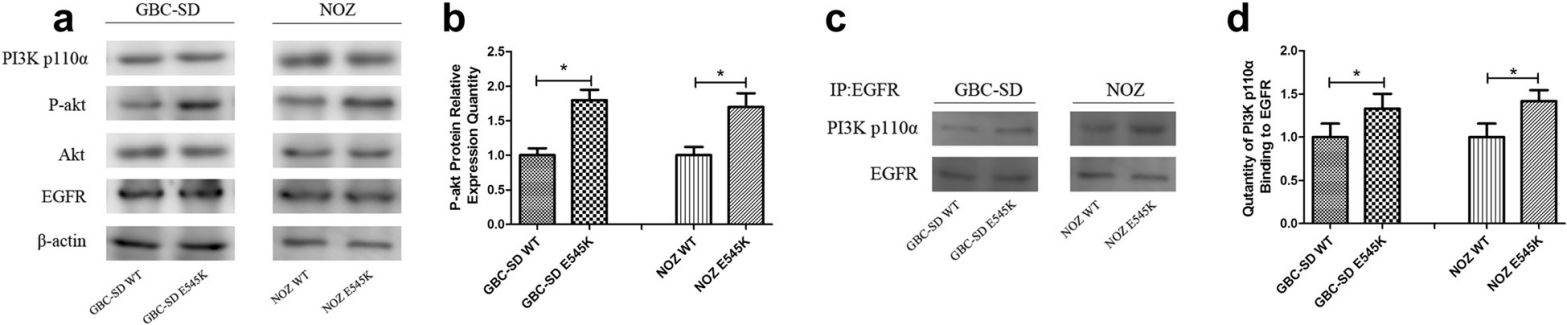
The E545K mutation enhanced PI3K p110α binding to EGFR. **a**, **b** After cellular starvation, EGF was added to the GBC-SD and NOZ cells. EGFR and key proteins in the PI3K-akt pathway were examined by Western blot assays. Representative chemiluminescent images of PI3K p110α, p-akt, akt and EGFR and the relative expression quantities of p-akt (mean ± SD, *n* = 3) are shown. **c**, **d** PI3K p110α binding to EGFR was examined by co-immunoprecipitation assays. Representative chemiluminescent images and the relative expression quantities of PI3K p110α binding to EGFR (mean ± SD, *n* = 3) are shown

To test this hypothesis, we assessed the amount of p110α binding to EGFR. After being starved, EGF was added to GBC-SD and NOZ cells and then cells were maintained to eliminate other confounding growth factor signals. We found activated Akt was increasing in E545K than in WT, although the expression of PI3K p110α and EGFR was similar in WT and E545K(shown in Fig. 5a, b). Meanwhile the quantity of PI3K p110α binding to EGFR in E545K was higher than the quantity in WT(shown in Fig. 5c, d). This shown E545K enhanced downstream Akt activity through enhancing PI3K p110α binding to EGFR.

### A selective PI3K p110α inhibitor suppressed GBC progression in both WT and E545K cells in vitro

Considering the increased activity of Akt in the E545K compared with WT cells and the worse prognosis of GBC patients harboring the E545K mutation, we sought to determine whether a PI3K inhibitor was effective in patients with the E545K mutation to satisfy the requirement of precision medicine. We selected A66, which is a widely used selective PI3K p110α inhibitor, to perform the follow-up experiments. We first measured the half maximal inhibitory concentration (IC_50_) of A66 in GBC cell lines and identified values of 15 μM/L A66 for the GBC-SD cells and 5 μM/L A66 for the NOZ cells in the in vitro experiments detailed below (Additional file 1: Figure S3a, b). IC_50_ of A66 was from 0.1 to 10 μM/L in various cells in published literatures [22–24].

**Fig. 6:**
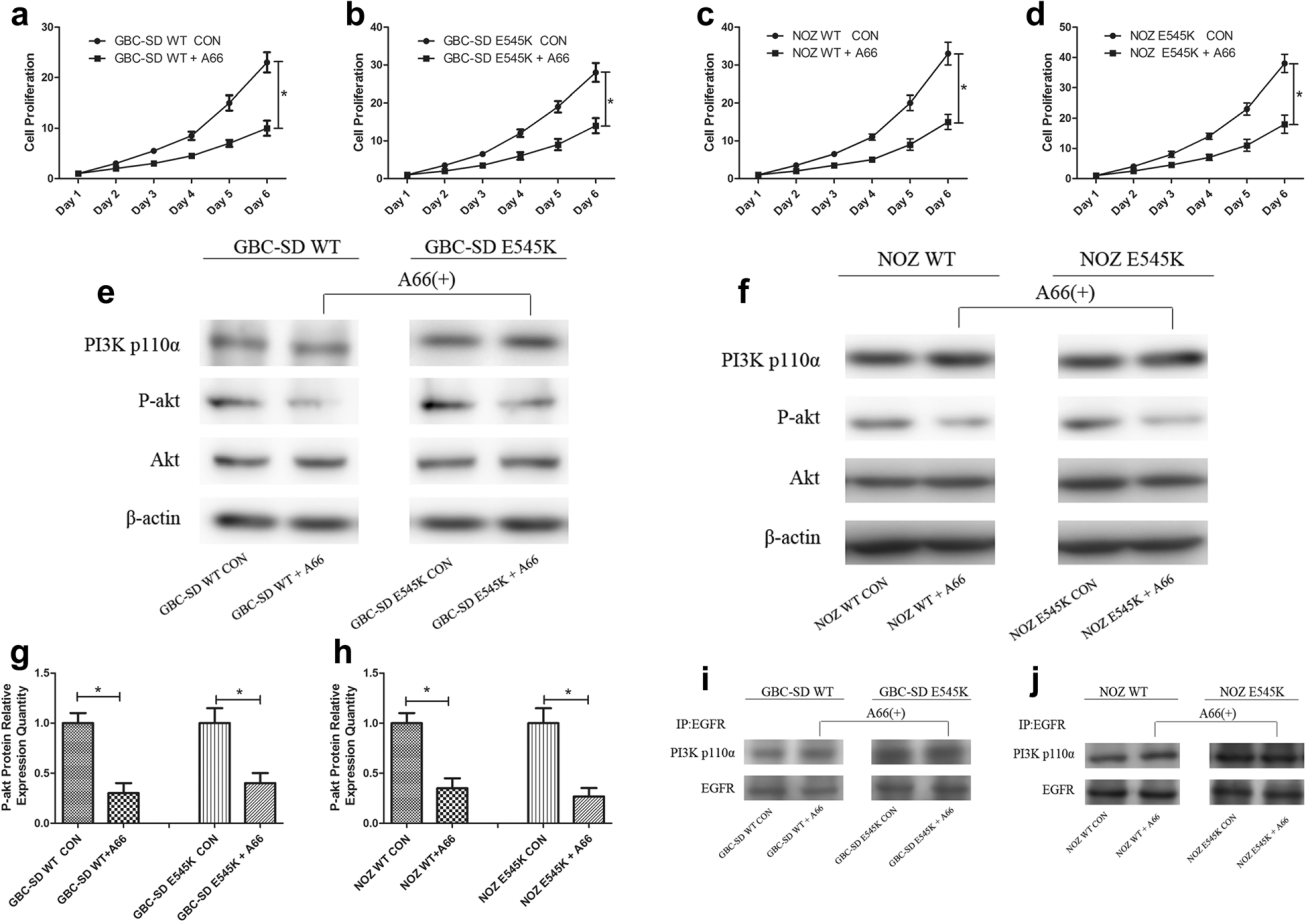
The selective PI3K p110α inhibitor suppressed GBC progression both in the WT and E545K cells in vitro. **a**-**d** After treated with (A66) or without (CON) 15 μM/L A66 for the GBC-SD cells and 5 μM/L A66 for the NOZ cells, Cell proliferation of WT or E545K GBC-SD and NOZ cells was evaluated using CCK8 cell viability assays. Cell proliferation was represented as cell quantity. Data are presented as mean ± SD (*n* = 5). **e**-**h** After treated with (A66) or without (CON) 15 μM/L A66 for the GBC-SD cells and 5 μM/L A66 for the NOZ cells for 24 h, key proteins in PI3K-akt pathway were examined by Western blot assays. Representative chemiluminescent images of PI3K p110α, p-akt and akt and the relative expression quantities of p-akt (mean ± SD, *n* = 3) are shown. **i**, **j** PI3K p110α binding to EGFR was examined by co-immunoprecipitation assays. Representative chemiluminescent images and the relative expression quantities of PI3K p110α binding to EGFR (mean ± SD, *n* = 3) are shown

We found that A66 not only suppressed the proliferation of WT GBC-SD and NOZ cells but also significantly suppressed the proliferation of E545K cells (Fig. 6a-d). Western blot analyses revealed that A66 significantly suppressed Akt activity in both WT and E545K cells but did not influence the expression of PI3K p110α (Fig. 6e-h). We also found that A66 did not influence the binding of PI3K p110α to EGFR (Fig. 6i, j). These results suggest that A66 suppressed GBC progression by converting PI3K p110α into an inactive form but did not down regulate the expression of PI3K p110α.

### The E545K mutation promoted GBC progression and the selective p110α inhibitor suppressed GBC progression in vivo

**Fig. 7:**
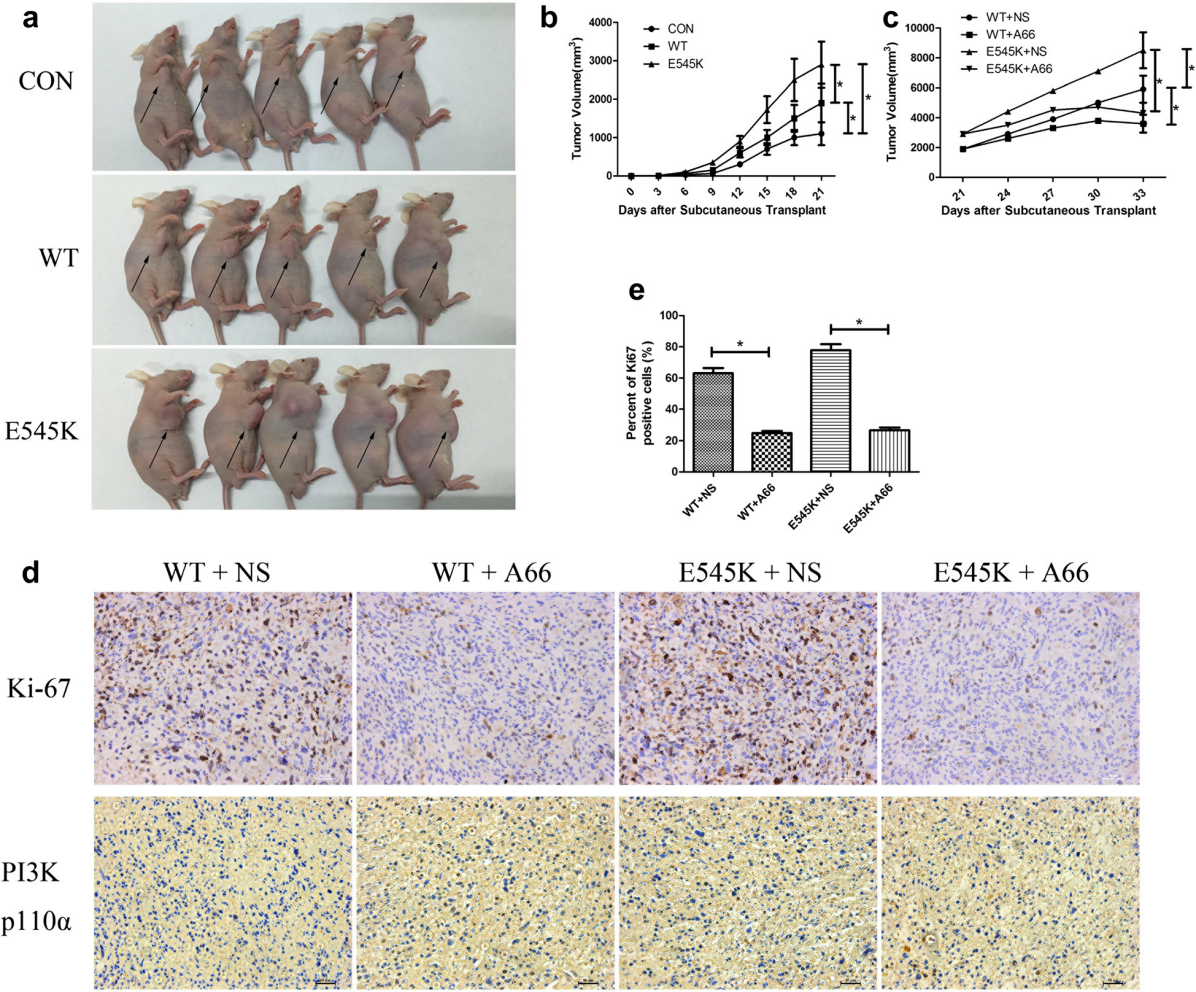
The E545K mutation promoted GBC progression and the selective p110α inhibitor suppressed GBC progression in vivo. **a** Mice bearing subcutaneous xenografts of CON, WT and E545K cells were anesthetized and photographed after three weeks. **b** The tumor volumes of CON, WT and E545K cell subcutaneous xenografts were measured every three days after inoculation. Data are presented as mean ± SD (*n* = 5). **c** Mice bearing WT and E545K cell subcutaneous xenografts were injected with 1 mg A66 or 100 μl NS every 3 days after inoculation for three weeks. Tumor volumes were measured every three days. Data are presented as mean ± SD (*n* = 5). **d** Representative photomicrographs of immunohistochemical staining for tumors resected from mice treated with A66 or NS are shown. **e** Percent of Ki67 positive cells is shown

We further used a transplantable subcutaneous mouse tumor model to explore the function of E545K in vivo. To obtain cell lines that stably expressed WT and E545K, we selected two cell lines from among 36 different monoclonal cells. Similar to the in vitro results, the E545K and WT lines significantly promoted tumor proliferation compared with the untreated cells (Fig. 7a, b). Moreover, the proliferation of the E545K line exceeded that of the WT line in vivo (Fig. 7a, b).

We also tested the effect of the selective PI3K p110α inhibitor in vivo. Three weeks after the transplantation of the tumor cells, the mice were injected with 1 mg A66 every 3 days for 3 weeks. We found that the tumor volumes and weights of the mice injected with A66 were obviously reduced compared with mice injected with normal saline (NS, Fig. 7c). Immunohistochemical staining for tumors resected from mice treated with A66 or NS was used to analyze tumor proliferation status. We found positive ratios of Ki67 in WT and E545K tumors treated with A66 were higher than in tumors treated with NS significantly (Fig. 7d, e), and representative photomicrographs were shown. Furthermore, we found expression quantity of PI3K p110α in WT and E545K tumors treated with A66 or NS was similar (Fig. 7d). These results demonstrate that the selective PI3K p110α inhibitor significantly suppressed GBC progression following the transplantation of both WT and E545K cells.

## Discussion

GBC is a highly fatal disease, thus, the mechanisms of GBC development and progression require urgent elucidation to identify new early diagnostic methods and novel drugs to improve the prognoses of patients with GBC [25, 26]. PI3Ks are widely known to play crucial roles in both normal cells and cancer cells [6, 7, 27]. Abnormal activation of the PI3K-akt pathway frequently leads to tumor development and progression [7, 27]. Numerous PI3K inhibitors have been tested in clinical trials, and inhibitor resistance has been observed. Hence, the unknown mechanism associated with the PI3K-akt pathway has aroused substantial interest among researchers. The PIK3CA E545K mutation has been identified in numerous cancers [17–21].

We followed up 130 patients with GBC for 36 months after surgery and found that the E545K mutation was associated with overall survival. The patients with the E545K mutation had significantly worse prognoses than the patients without this mutation. We did not identify any significant associations of the PIK3CA E545K mutation with the clinical data. The possible cause is that the sample size was not sufficiently large to reveal such relationships.

The knockdown of PIK3CA significantly suppressed GBC cell proliferation, migration, invasion and clone formation, and the overexpression of PIK3CA obviously promoted these processes in vitro. These findings conclusively demonstrated the vital function of PIK3CA in GBC cells. Compared with the WT PIK3CA, the PIK3CA with the E545K mutation exhibited stronger effects on cell proliferation, migration, invasion and clone formation. Similarly, the E545K mutation exhibited a stronger tumor proliferation-enhancing effect than the WT in vivo. These findings are in agreement with discoveries related to other tumors [8, 28]. We found that the phosphorylated form of akt was elevated in the E545K mutants compared with the WTs, but the expression of PIK3CA was similar in the WT and E545K cells. PI3K p110α (encoded by PIK3CA) and PI3K p110β (encoded by PIK3CB) play similar roles in the PI3K-akt pathway, but the activity of p110α is greater than that of p110β [24]. Therefore, we hypothesized that the E545K mutation could enhance the ability of p110α to bind to upstream RTKs. We subsequently quantified the p110α binding to EGFR (which is one of the most important RTKs) in WT and E545K cell lines using co-immunoprecipitation (CO-IP). Interestingly, we observed greater binding of p110α to EGFR in the E545K cells than in WT cells, although p110α expression was similar in the WT and E545K cell lines. Together, these results demonstrated that the E545K mutation of PIK3CA promotes tumor progression in GBC via enhancement of the binding to EGFR. The E545K and H1047R mutations of PIK3CA increase PIP_3_ levels, activate akt signaling and induce cellular transformation [7, 29, 30], but the exact molecular mechanism by which these mutations activate p110α has not been determined. Some studies have demonstrated that, the regulatory p85 subunit stabilizes the catalytic p110α subunit and inhibits its enzymatic activity in the basal state [31]. The p110α subunit contains an N-terminal adaptor-binding domain, a Ras-binding domain, a C2 domain, a helical domain, a catalytic domain, moreover, the helical domain is related to the interaction with p85 [32]. The mechanism that is currently thought to explain the oncogenic effect of the E545K mutation is that the helical domain mutation weakens p110α’s interactions with the p85 regulatory subunits, which attenuates the inhibitory effect of p85 and thus increases the enzymatic activity of p110α [33, 34] Moreover, another study suggested that the weakened p110α-p85 interaction caused by the mutation is not sufficient for the p110α mutant proteins to exert their oncogenic functions and provided several pieces of evidence that the p110α E545K-IRS1 interaction plays a critical role in tumorigenesis [28]. Our study advanced the explanation of the oncogenic effect of the E545K mutation by demonstrating that this mutation promotes tumor progression through enhanced binding to EGFR in GBC.

Because patients with the E545K mutation have worse prognoses than those without this mutation, we believe that the needs of patients with E545K mutation for new effective drugs is greater than that of patients without the mutation. Although numerous PI3K inhibitors have been tested in clinical trials, no drugs are available that target the E545K mutation. We are convinced that the efficacy of these drugs should be confirmed in clinical trials involving patients with the E545K mutation based on the precision medicine perspective. Some studies have reported that selective inhibitors may have better application potentials due to their lower toxicities [8]. Therefore, we tested a selective inhibitor of PI3K p110α (A66) in vivo and in vitro. Interestingly, we found that A66 significantly suppressed tumor proliferation in both WT and E545K cells lines.

In this study, we found that patients with E545K mutation had worse prognoses than those without this mutation and confirmed that the E545K significantly promoted tumor progression both in vitro and in vivo. Excitingly, the PI3K inhibitor was effective against the E545K mutation. Considering the lethality of GBCs and highly malignant effect of PIK3CA E545K, we believe that the early use of effective drugs following gene sequencing may represent an efficacious method for improving the prognoses of GBC patients harboring the PIK3CA E545K mutation.

## Conclusions

The prognoses of patients with E545K mutation were worse than patients without this mutation. The E545K mutation promoted GBC progression through enhanced binding to EGFR and activating downstream akt activity. The PI3K selective inhibitor, A66, suppressed gallbladder carcinoma proliferation.

## Methods

### Study population

The GBC tissue specimens were obtained from 130 patients who underwent cholecystectomies from 2008 and 2013 in the Department of General Surgery, Xinhua Hospital. The sequencing technology was provided by BioSune (Shanghai, China). The patients follow-ups were performed via telephone once every three months. This study was approved by the ethics committee of Xinhua Hospital, School of Medicine, Shanghai Jiao Tong University. All patients provided written informed consent.

### Cell lines and culture

The human gallbladder cancer cell lines GBC-SD, NOZ, SGC-996, OCUG, EHGB-1 and EHGB-2 were purchased from Shanghai Institute of Cell Biology, Chinese Academy of Sciences (CAS, Shanghai, China). The GBC-SD cells were grown in high-glucose DMEM (Gibco, Grand Island, NY, USA) supplemented with 10 % fetal bovine serum (Gibco) and 1 % penicillin-streptomycin(Hyclone, Logan, UT, USA). The NOZ cells were grown in William’s medium (Gibco) supplemented with 10 % fetal bovine serum (Gibco) and 1 % penicillin-streptomycin (Hyclone). The SGC cells were grown in 1640 medium (Gibco) supplemented with 10 % fetal bovine serum (Gibco) and 1 % penicillin-streptomycin (Hyclone), The OCUG, EHGB-1 and EHGB-2 cells were grown in high-glucose DMEM (Gibco) supplemented with 15 % fetal bovine serum (Gibco) and 1 % penicillin-streptomycin (Hyclone). All cells lines were maintained at 37 °C in a humidified atmosphere with 5 % CO_2_. The A66 used in the in vitro and in vivo experiments was purchased from Selleck Chemicals (Houston, USA). Before conducting the CO-IP experiments, the GBC-SD and NOZ cells were starved for 12 h and 1 ng/mL EGF (Sangon Biotech, Shanghai, China) was added to the cell culture medium. The cells were maintained for 12 h to eliminate other confounding growth factor signals.

### RT-PCR, Q-PCR and sequencing

Total RNA was extracted from the cultured GBC-SD, NOZ, SGC-996, OCUG, EHGB-1, and EHGB-2 cells or tissue samples with Trizol reagent (Takara, Shiga, Japan). cDNA was synthesized from 1 μg of total RNA using random primers and M-MLV Reverse Transcriptase (Takara). The RNA expression quantities were measured by RT-PCR using the SYBR-Green method (Takara) on a StepOnePlus system (Thermo Fisher Scientific, USA) according to the manufacturer’s instructions. The relative expression levels of the target genes were calculated by as 2^-ΔCT^ (ΔCT = C_T_^PIK3CA^ -C_T_^GADPH^) and normalized to the relative expressions detected in the corresponding control cells, which were defined as 1.0. In the present study, the expression levels (defined as the fold changes) of PIK3CA were calculated as 2^-ΔΔCT^(ΔΔCT = ΔCT_sample1_-ΔCT_sample2_). The sequencing service was provided by BioSune (Shanghai, China).

The RT-PCR primer sequences were as follows:

F-primer: 5′-AGAAGATTTGCTGAACCCTATTGG-3′

R-primer: 5′-CACTGACATATCTGGGAACTTTACC-3′

The sequencing Primer sequences were as follows:

F-primer: 5′-ATACATCTGGGCTACTTC-3′

R-primer: 5′-ACCCTATTGGTGTTACTG-3′

### Cell transfections with siRNA or plasmid

The siRNAs were synthesized by Biomics (Nantong, China). The siRNA sequences were as follows:

hs-PIK3CA siRNA1 positive-sense strand: 5′-GACACUCUAGUAUCUGGAAdTdT-3′

anti-sense strand: 5′-UUCCAGAUACUAGAGUGUCdTdT-3′

hs-PIK3CA siRNA2 positive-sense strand: 5′-CAGAGUUACUGUUUCAGAAdTdT-3′

anti-sense strand: 5′-UUCUGAAACAGUAACUCUGdTdT-3′

hs-PIK3CA siRNA3 positive-sense strand: 5′-GAGAUGUGUUACAAGGCUUdTdT-3′

anti-sense strand: 5′-AAGCCUUGUAACACAUCUCdTdT-3′

The PIK3CA-WT(wile-type) plasmid and the PIK3CA-E545K mutation plasmid were gifts from Bert Vogelstein [17] (Addgene plasmid #16643 and #16642, respectively). siRNA or plasmids were transfected into the cells using Lipofectamine 2000 (Thermo Fisher Scientific) according to the manufacturer’s instructions.

### Western blot and Co-IP analyses

The GBC-SD and NOZ cells were collected with the culture medium into centrifuge tubes. After centrifugation at 1500 r/min for 3 min, the cells were washed twice with cold phosphate-buffered saline (PBS). Then, the cells were lysed with RIPA buffer (Beyotime Institute of Biotechnology, Beijing, China) and protease inhibitor (Roche Applied Science, Indianapolis, IN, USA) at 4 °C for 5 min. After centrifugation at 14,000 r/min at 4 °C for 5 min, the protein concentration was measured using a bicinchoninic acid (BCA) assay kit (Beyotime, Shanghai, China). Equal amounts of protein (40 μg/lane) from the different samples were separated by 10 % sodium dodecyl sulfate polyacrylamide gel electrophoresis (SDS-PAGE) and then electrophoretically transferred to nitrocellulose membranes (Millipore, Bedford, MA, USA). The membranes were blocked with 5 % skim milk for 2 h and then incubated with the primary antibodies against PI3K p110α (CST, Cell Signaling Technology, USA), Bcl-2(Proteintech), Bax(Proteintech), cyclin D1(Proteintech), β-actin(Proteintech), vimentin(Proteintech), E-cadherin(Proteintech), Akt and P-akt (Proteintech) (1:1000) at 4 °C overnight. The membranes were incubated with the secondary antibodies (horseradish peroxidase HRP-conjugated goat anti-rabbit/anti-mouse IgG 1:5000; Abcam, Cambridge, UK) for 2 h after washing three times with TBST buffer. The bands were visualized using a chemiluminescent detection kit (Rockford, IL, USA) and photographed using a Gel Doc 2000 (BioRad, Hercules,CA, USA). The immunoprecipitations were set up as follows: Lysates containing 0.5 mg of total protein were incubated with 1 1 μL of EGFR antibody(CST). All immunoprecipitations were performed overnight, and protein A/G sepharose (Santa Cruz Biotechnology) was added for 2 h. The beads were then collected by centrifugation at 1000 r/min, followed by three washes with lysis buffer. The resulting immunoprecipitates were subjected to Western blot analyses.

### Cell proliferation assay

The proliferations of the GBC-SD and NOZ cells lines treated with siRNA, negative control (NC) siRNA (random null siRNA sequence), CON (only Lipofectamine 2000), WT (PIK3CA-WT plasmid), E545K (PIK3CA-E545K plasmid) and vector (empty vector) were measured using a Cell Counting Kit-8 (CCK8, Dojindo, Japan) assay according to the manufacturer’s instructions. The GBC-SD (8 × 10^4^) and NOZ (5 × 10^4^) cells were seeded into 6-well plates (Corning, NY, USA) and incubated overnight. The cells were then transfected with siRNA, NC, CON, WT, E545K or vector. The cells were maintained for 12 h and then seeded into 96-well plates (500 cells in each well). The cells were maintained for 6, 24, 48, 72, 96 and 120 h, and CCK-8 was then added to each well, and cells were incubated at 37 °C for 3 h. The optical densities (ODs) were measured at a wavelength of 450 nm with a microplate reader (QuantBio Tek Instruments, Winooski, VT, USA). The results are represented as averages of five different wells. Cell proliferation was measured according to the following the formula: cell proliferation =  (OD_Day n_/OD_Day1_)*100 %.

### Cell migration and invasion assays

Cell migration and invasion were examined using transwell filters (BD Biosciences, Franklin Lakes, NJ). GBC-SD and NOZ cells were treated with siRNA, NC, CON, WT, E545K or vector same as described above. GBC-SD (2 × 10^4^) and NOZ (1.5 × 10^4^) cells in 200 μL serum-free DMED medium or William’s medium were added to the upper chamber, which contained an uncoated or Matrigel (BD Biosciences)-coated membrane. The lower chamber was filled with 500 μL of basal medium with 10 % fetal bovine serum. After incubation at 37 °C in a humidified 5 % CO_2_ incubator for 18 h for the siRNA knockdown experiments or 12 h for the plasmid overexpression experiments in the uncoated transwells and 24 h for the siRNA knockdown experiments or 18 h for the plasmid overexpression experiments in the Matrigel-coated transwells, the cells that migrated to the lower compartment were fixed with 4 % paraformaldehyde for 20 min and stained with 0.1 % crystal violet (Sigma-Aldrich) for 30 min. The migrated or invaded cells were counted in five fields (above, below, left, right and middle of the well) in each well. Imaging and cell counting were performed under a microscope (Leica, Wetzlar, Germany).

### Colony formation assay

GBC-SD and NOZ cells were treated with siRNA, NC, CON, WT, E545K or vector as what described above. The cells were dispersed as single cell suspensions, and 500 cells per well were planted into 6-well plates. The cells were cultured (5 days for the NOZ cells and 14 days for the GBC-SD cells) to allow for colony formation. Subsequently, the cells were fixed with 4 % paraformaldehyde for 20 min and stained with 0.1 % crystal violet for 30 min. The plates were air-dried after washing and the stained colonies were photographed using a microscope. The total number of colonies (>50 cells/colony) was counted.

### Screening of the stable expression cell lines

The PIK3CA-WT and PIK3CA-E545K mutation plasmids were transfected into NOZ cells with Lipofectamine 2000 (Thermo Fisher Scientific). Cell culture medium with 1000 μg/mL G418 was added and then replaced with fresh culture medium with 500 μg/mL G418 every three days. The cells were cultured for two weeks at 37 °C in a humidified atmosphere with 5 % CO_2_. The cells were diluted to single cells and planted into 96-well plates. Monoclones were selected for amplification and then assessed by Western blot assays using anti-flag antibody (Sigma-Aldrich, USA).

### In vivo subcutaneous xenograft analysis

The animal use and the experiment protocol were approved by the Institutional Animal Care and Use Committee of Xinhua Hospital, School of Medicine, Shanghai Jiao Tong University. Subcutaneous xenograft models were established as previously described [35]. Nude mice were randomly divided into control, WT and E545K mutation groups that received subcutaneous injection inoculations with 1 × 10^6^ NOZ, NOZ-WT and NOZ-E545K cells, respectively. The tumor volumes were measured using Vernier calipers once every three days, and the tumor volumes were calculated according to the following formula: tumor volume (mm3) = L × W^2^ (where L and W represent the length and width of the tumor, respectively). On day 21, the mice were anesthetized and photographed. Afterwards, the mice in the control group were sacrificed. The WT group and the E545K mutation group were randomly divided into A66 group and normal saline groups. The mice in the A66 group were injected with 1 mg A66 once every three days through the tail vein for three weeks, and the mice in normal saline group were injected with same volume of normal saline. The tumor volumes were measured once every three days as previously described. On day 42, the mice were sacrificed, and the tumor tissues were removed and weighed.

### Immunohistochemistry(IHC) assays

Three weeks after treated with A66 or NS, the mice were sacrificed and the tumors were dissected out. The PI3K p110α expression and proliferative index of Ki-67 was evaluated in xenograft tumors by immunohistochemical staining (IHC). IHC staning was performed as previously described [2].

### Statistical analysis

SPSS 18.0 software was used to perform the statistical analyses. All values are expressed as the mean ± the standard deviation (SD) unless otherwise stated. Student’s t-tests were performed to compare the differences between the different experiment groups unless otherwise stated. *P*-values less than 0.05 were considered statistically significant (*P* < 0.05 is denoted by *).

## Additional file

Additional file 1: Table S1. The clinical data for the patients in the study. **Figure S1**. (a) Expression of PIK3CA in NOZ, GBC-SD, SGC-996, OCUG, EHGB-1 and EHGB-2 cell lines was measured by RT-PCR. Relative expression quantity was shown(mean ± SD, *n* = 5). (b, c) Three different siRNAs werer designed to knock down PIK3CA in GBC-SD and NOZ. Expression of PIK3CA was measured by RT-PCR. Relative expression quantity was shown(mean ± SD, *n* = 5). **Figure S2**. (a) RT-PCR was used to measure the relative expression of PIK3CA of GBC-SD and NOZ after be transfected with WT and E545K plasmid. Data are presented as mean ± SD (*n* = 5). (b, c) Expression of PI3K p110α was examined by Western-Blot assay. Representative chemiluminescent images and the relative expression quantity (mean ± SD, *n* = 3). **Figure S3**. (a, b) GBC-SD and NOZ cells treated with different concentration of A66 were evaluated by CCK8 cell viability assay for 3 days. Data are presented as mean ± SD (*n* = 5). (DOCX 482 kb)
