# Supplementary material for: The E545K mutation of PIK3CA promotes gallbladder carcinoma progression through enhanced binding to EGFR
Source: J Exp Clin Cancer Res. 2016 Jun 18;35:97. doi: 10.1186/s13046-016-0370-7 (PMC4912708; doi:10.1186/s13046-016-0370-7)
Supplement: Additional file 1: Table S1. — The clinical data for the patients in the study. Figure S1. (a) Expression of PIK3CA in NOZ, GBC-SD, SGC-996, OCUG, EHGB-1 and EHGB-2 cell lines was measured by RT-PCR. Relative expression quantity was shown(mean ± SD, n = 5). (b, c) Three different siRNAs werer designed to knock down PIK3CA in GBC-SD and NOZ. Expression of PIK3CA was measured by RT-PCR. Relative expression quantity was shown(mean ± SD, n = 5). Figure S2. (a) RT-PCR was used to measure the relative expression of PIK3CA of GBC-SD and NOZ after be transfected with WT and E545K plasmid. Data are presented as mean ± SD (n = 5). (b, c) Expression of PI3K p110α was examined by Western-Blot assay. Representative chemiluminescent images and the relative expression quantity (mean ± SD, n = 3). Figure S3. (a, b) GBC-SD and NOZ cells treated with different concentration of A66 were evaluated by CCK8 cell viability assay for 3 days. Data are presented as mean ± SD (n = 5). (DOCX 482 kb) [file 13046_2016_370_MOESM1_ESM.docx]

The E545K mutation of PIK3CA promotes gallbladder carcinoma progression through enhanced binding to EGFR

Shuai Zhao^+^, Yang Cao^+^, Shi-bo Liu^+^, Xu-an Wang, Run-fa Bao, Yi-jun Shu, Yun-ping Hu, Yi-jian Zhang, Lin Jiang, Fei Zhang, Hai-bin Liang, Huai-feng Li, Qiang Ma, Yi Xu, Zheng Wang, Yi-chi Zhang, Lei Chen*, Jian Zhou*, Ying-bin Liu*

1 Department of General Surgery and Laboratory of General Surgery, Xinhua Hospital, Affiliated to Shanghai Jiao Tong University, School of Medicine, Shanghai 200092

2 Institute of Biliary Tract Diseases Research, Shanghai Jiao Tong University School of Medicine, Shanghai 200092

Supplemental Table 1: The clinical data for the patients in the study

| Sample | Gender | Age | Lymph node metastasis | TNM stage | Margin status | Jaundice | Survival status | Months to last follow up | PIK3CA mutation |
| --- | --- | --- | --- | --- | --- | --- | --- | --- | --- |
| GBCS1 | M | 61 | NO | IIIA | R1 | YES | Deceased | 10.3 | NO |
| GBCS2 | M | 58 | NO | IIIA | R0 | NO | Living | 36 | NO |
| GBCS3 | F | 62 | YES | IIIB | R1 | NO | Deceased | 4.1 | NO |
| GBCS4 | F | 57 | NO | IIIA | R0 | NO | Deceased | 8.3 | YES |
| GBCS5 | M | 56 | YES | IIIB | R1 | NO | Deceased | 7 | NO |
| GBCS6 | M | 76 | NO | IIIA | R1 | NO | Deceased | 5 | NO |
| GBCS7 | M | 62 | NO | IIIA | R0 | YES | Deceased | 20 | NO |
| GBCS8 | F | 67 | YES | IIIB | R1 | NO | Living | 36 | NO |
| GBCS9 | M | 72 | NO | IIIA | R0 | NO | Living | 22 | NO |
| GBCS10 | F | 78 | NO | II | R0 | YES | Living | 15 | NO |
| GBCS11 | M | 86 | YES | IVB | R1 | YES | Deceased | 2 | NO |
| GBCS12 | F | 42 | YES | IIIB | R1 | NO | Living | 36 | NO |
| GBCS13 | F | 65 | YES | IIIB | R1 | NO | Deceased | 2.5 | NO |
| GBCS14 | F | 47 | NO | IVB | R1 | NO | Deceased | 18 | NO |
| GBCS15 | F | 42 | NO | IIIA | R0 | YES | Deceased | 3 | NO |
| GBCS16 | F | 61 | YES | IIIB | R0 | NO | Deceased | 18 | NO |
| GBCS17 | M | 37 | NO | IIIA | R0 | YES | Deceased | 2.6 | NO |
| GBCS18 | M | 65 | NO | IIIA | R1 | NO | Deceased | 10 | NO |
| GBCS19 | F | 77 | NO | II | R0 | NO | Living | 13 | NO |
| GBCS20 | M | 78 | NO | IIIA | R0 | NO | Deceased | 6 | NO |
| GBCS21 | M | 60 | NO | IIIA | R1 | NO | Deceased | 8 | NO |
| GBCS22 | F | 81 | NO | IIIA | R0 | NO | Deceased | 7.6 | NO |
| GBCS23 | F | 61 | NO | IIIA | R1 | NO | Deceased | 8 | NO |
| GBCS24 | F | 51 | NO | IIIA | R0 | NO | Living | 10 | NO |
| GBCS25 | F | 48 | NO | IIIA | R0 | NO | Deceased | 7 | NO |
| GBCS26 | F | 48 | NO | IIIA | R0 | NO | Deceased | 11 | NO |
| GBCS27 | F | 77 | NO | IIIA | R0 | NO | Deceased | 6 | YES |
| GBCS28 | M | 74 | NO | IIIA | R0 | YES | Deceased | 9 | NO |
| GBCS29 | M | 68 | NO | IIIA | R0 | NO | Deceased | 9 | NO |
| GBCS30 | M | 55 | NO | IIIA | R0 | NO | Deceased | 9 | YES |
| GBCS31 | M | 68 | YES | IIIB | R1 | YES | Deceased | 8 | NO |
| GBCS32 | F | 46 | NO | IIIA | R0 | YES | Deceased | 6 | NO |
| GBCS33 | F | 59 | NO | IIIA | R0 | NO | Deceased | 12 | NO |
| GBCS34 | M | 66 | NO | IIIA | R0 | NO | Living | 36 | NO |
| GBCS35 | F | 57 | NO | IIIA | R1 | NO | Deceased | 2 | NO |
| GBCS36 | F | 59 | NO | IIIA | R1 | NO | Deceased | 8 | NO |
| GBCS37 | M | 45 | YES | IVB | R1 | NO | Deceased | 8.3 | NO |
| GBCS38 | M | 61 | NO | IIIA | R0 | NO | Deceased | 21 | NO |
| GBCS39 | F | 54 | YES | IIIB | R1 | NO | Deceased | 13 | NO |
| GBCS40 | M | 58 | YES | IVB | R1 | YES | Deceased | 5 | NO |
| GBCS41 | F | 81 | YES | IIIB | R1 | YES | Deceased | 7 | NO |
| GBCS42 | F | 56 | YES | IVB | R1 | YES | Deceased | 4 | NO |
| GBCS43 | F | 46 | YES | IVB | R0 | YES | Deceased | 7 | NO |
| GBCS44 | F | 61 | NO | IIIA | R0 | NO | Deceased | 23 | NO |
| GBCS45 | F | 64 | NO | IIIA | R0 | YES | Deceased | 14 | NO |
| GBCS46 | F | 55 | YES | IVB | R1 | YES | Deceased | 5 | NO |
| GBCS47 | M | 59 | NO | II | R0 | YES | Deceased | 9 | NO |
| GBCS48 | F | 54 | YES | IIIB | R1 | NO | Deceased | 7 | NO |
| GBCS49 | M | 50 | YES | IIIB | R1 | YES | Deceased | 9.5 | NO |
| GBCS50 | F | 65 | YES | IIIB | R1 | YES | Deceased | 2.5 | NO |
| GBCS51 | M | 48 | YES | IIIB | R0 | NO | Deceased | 8 | NO |
| GBCS52 | F | 47 | YES | IIIB | R1 | NO | Deceased | 8 | NO |
| GBCS53 | M | 49 | NO | IIIA | R0 | NO | Living | 12 | NO |
| GBCS54 | F | 73 | NO | IIIA | R0 | NO | Deceased | 7 | NO |
| GBCS55 | F | 74 | YES | IIIB | R1 | NO | Deceased | 8 | NO |
| GBCS56 | F | 53 | YES | IVB | R1 | NO | Deceased | 12 | NO |
| GBCS57 | F | 79 | YES | IIIB | R1 | NO | Deceased | 6.5 | NO |
| GBCS58 | F | 46 | NO | IVB | R0 | NO | Deceased | 2.8 | NO |
| GBCS59 | M | 77 | NO | II | R0 | NO | Living | 18 | NO |
| GBCS60 | M | 39 | NO | IIIA | R1 | YES | Deceased | 6.8 | NO |
| GBCS61 | M | 55 | YES | IVB | R0 | NO | Deceased | 3.5 | NO |
| GBCS62 | F | 58 | NO | IIIA | R0 | NO | Living | 36 | NO |
| GBCS63 | M | 67 | YES | IIIB | R0 | YES | Deceased | 7.7 | NO |
| GBCS64 | F | 61 | YES | IIIB | R1 | YES | Deceased | 9.5 | NO |
| GBCS65 | F | 71 | NO | IIIA | R0 | NO | Deceased | 17 | NO |
| GBCS66 | F | 53 | NO | IIIA | R0 | NO | Deceased | 13 | NO |
| GBCS67 | M | 66 | NO | II | R0 | YES | Living | 13 | NO |
| GBCS68 | M | 62 | NO | IIIA | R0 | NO | Living | 36 | NO |
| GBCS69 | F | 43 | NO | IVB | R1 | NO | Deceased | 6 | NO |
| GBCS70 | F | 55 | YES | IIIB | R1 | NO | Deceased | 4.3 | YES |
| GBCS71 | M | 84 | YES | IVB | R0 | NO | Deceased | 7.6 | NO |
| GBCS72 | F | 74 | NO | IIIA | R1 | NO | Deceased | 9.5 | NO |
| GBCS73 | F | 66 | NO | IIIB | R1 | NO | Deceased | 3.7 | NO |
| GBCS74 | M | 69 | YES | IIIA | R0 | NO | Deceased | 14 | NO |
| GBCS75 | M | 57 | NO | IIIA | R0 | NO | Living | 16 | NO |
| GBCS76 | F | 54 | YES | IIIB | R1 | YES | Deceased | 5 | NO |
| GBCS77 | M | 50 | NO | IIIA | R0 | NO | Living | 36 | NO |
| GBCS78 | M | 48 | YES | IVB | R1 | YES | Deceased | 5.3 | NO |
| GBCS79 | M | 68 | NO | IIIA | R0 | NO | Deceased | 18 | NO |
| GBCS80 | M | 71 | NO | IIIA | R0 | NO | Deceased | 11.2 | NO |
| GBCS81 | F | 72 | NO | IIIA | R0 | NO | Deceased | 9.6 | NO |
| GBCS82 | F | 68 | YES | IVB | R1 | YES | Deceased | 8.8 | NO |
| GBCS83 | M | 62 | NO | IIIA | R1 | NO | Deceased | 17 | NO |
| GBCS84 | M | 51 | NO | IIIA | R0 | NO | Living | 36 | NO |
| GBCS85 | F | 73 | YES | IIIB | R1 | YES | Deceased | 10.7 | NO |
| GBCS86 | F | 56 | NO | IIIA | R0 | NO | Living | 36 | NO |
| GBCS87 | M | 63 | YES | IVB | R1 | YES | Deceased | 3 | YES |
| GBCS88 | F | 69 | NO | IIIA | R1 | NO | Deceased | 15 | NO |
| GBCS89 | M | 49 | NO | IIIA | R0 | NO | Living | 36 | NO |
| GBCS90 | F | 66 | NO | IIIB | R1 | NO | Deceased | 17 | YES |
| GBCS91 | M | 53 | NO | IIIA | R1 | NO | Deceased | 11 | NO |
| GBCS92 | M | 60 | NO | IIIA | R0 | NO | Deceased | 16 | NO |
| GBCS93 | F | 67 | NO | IIIA | R0 | NO | Deceased | 8.4 | NO |
| GBCS94 | M | 57 | NO | IIIA | R0 | YES | Deceased | 11.8 | NO |
| GBCS95 | F | 49 | YES | IVB | R1 | NO | Deceased | 6.7 | NO |
| GBCS96 | M | 69 | NO | IIIA | R1 | NO | Deceased | 7.2 | NO |
| GBCS97 | F | 55 | NO | IIIB | R0 | YES | Living | 16 | NO |
| GBCS98 | F | 78 | YES | IIIA | R1 | YES | Deceased | 8.4 | NO |
| GBCS99 | M | 57 | NO | IIIA | R0 | NO | Living | 17 | NO |
| GBCS100 | F | 71 | YES | IVB | R1 | NO | Deceased | 5.5 | NO |
| GBCS101 | F | 67 | NO | IIIA | R0 | YES | Deceased | 7.3 | NO |
| GBCS102 | M | 63 | NO | IIIA | R1 | NO | Deceased | 14 | NO |
| GBCS103 | F | 70 | NO | IIIB | R0 | NO | Deceased | 7.9 | NO |
| GBCS104 | M | 59 | NO | IIIA | R1 | YES | Deceased | 15 | NO |
| GBCS105 | F | 48 | NO | II | R0 | NO | Living | 36 | NO |
| GBCS106 | F | 59 | NO | IIIA | R0 | NO | Deceased | 19 | NO |
| GBCS107 | F | 57 | NO | IIIA | R1 | YES | Deceased | 6.1 | NO |
| GBCS108 | M | 68 | NO | II | R1 | NO | Deceased | 11 | NO |
| GBCS109 | M | 58 | YES | IIIA | R1 | YES | Deceased | 10.5 | NO |
| GBCS110 | M | 72 | NO | IIIB | R1 | NO | Deceased | 8 | NO |
| GBCS111 | F | 63 | NO | IIIB | R0 | NO | Deceased | 9 | NO |
| GBCS112 | M | 54 | NO | IIIA | R1 | NO | Deceased | 6 | NO |
| GBCS113 | F | 52 | NO | IIIA | R0 | NO | Living | 36 | NO |
| GBCS114 | M | 72 | YES | IVB | R0 | YES | Deceased | 4.8 | YES |
| GBCS115 | F | 55 | NO | IIIA | R1 | NO | Deceased | 5.1 | NO |
| GBCS116 | M | 50 | NO | IIIA | R0 | NO | Living | 36 | NO |
| GBCS117 | M | 63 | NO | IIIA | R0 | NO | Deceased | 13 | NO |
| GBCS118 | F | 60 | YES | IVB | R1 | YES | Deceased | 4.4 | NO |
| GBCS119 | F | 57 | NO | IIIA | R0 | YES | Deceased | 9.3 | NO |
| GBCS120 | M | 62 | NO | IIIA | R0 | NO | Deceased | 12 | NO |
| GBCS121 | M | 77 | NO | II | R0 | NO | Living | 36 | NO |
| GBCS122 | M | 63 | YES | IIIA | R1 | NO | Deceased | 5.9 | NO |
| GBCS123 | F | 79 | NO | IIIA | R0 | YES | Deceased | 9.3 | NO |
| GBCS124 | M | 52 | NO | IIIA | R0 | NO | Living | 23 | NO |
| GBCS125 | F | 76 | YES | IIIB | R0 | NO | Deceased | 8.7 | YES |
| GBCS126 | F | 59 | NO | IIIA | R0 | NO | Deceased | 15 | NO |
| GBCS127 | F | 60 | YES | IVB | R1 | NO | Deceased | 5.9 | NO |
| GBCS128 | M | 56 | NO | IIIA | R0 | YES | Deceased | 14 | NO |
| GBCS129 | M | 61 | NO | IIIA | R0 | NO | Deceased | 13 | NO |
| GBCS130 | M | 63 | YES | IVB | R1 | NO | Deceased | 5 | NO |

Supplemental Figure 1:


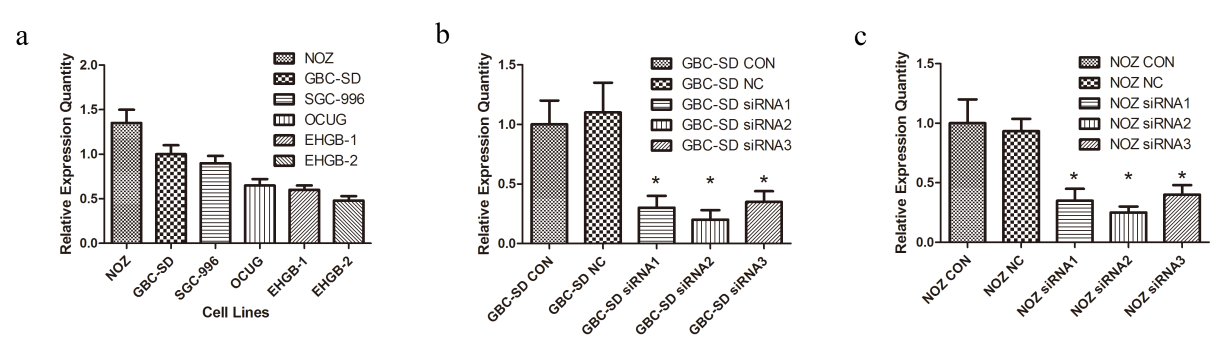


(a) Expression of PIK3CA in NOZ, GBC-SD, SGC-996, OCUG, EHGB-1 and EHGB-2 cell lines was measured by RT-PCR. Relative expression quantity was shown(mean ± SD, n=5). (b, c) Three different siRNAs werer designed to knock down PIK3CA in GBC-SD and NOZ. Expression of PIK3CA was measured by RT-PCR. Relative expression quantity was shown(mean ± SD, n=5).

Supplemental Figure 2:


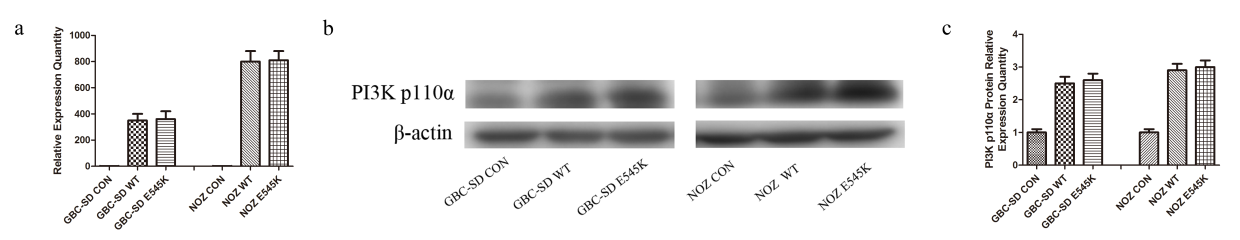


(a) RT-PCR was used to measure the relative expression of PIK3CA of GBC-SD and NOZ after be transfected with WT and E545K plasmid. Data are presented as mean ± SD (n = 5). (b, c) Expression of PI3K p110α was examined by Western-Blot assay. Representative chemiluminescent images and the relative expression quantity (mean ± SD, n = 3)

Supplemental Fiugre 3:


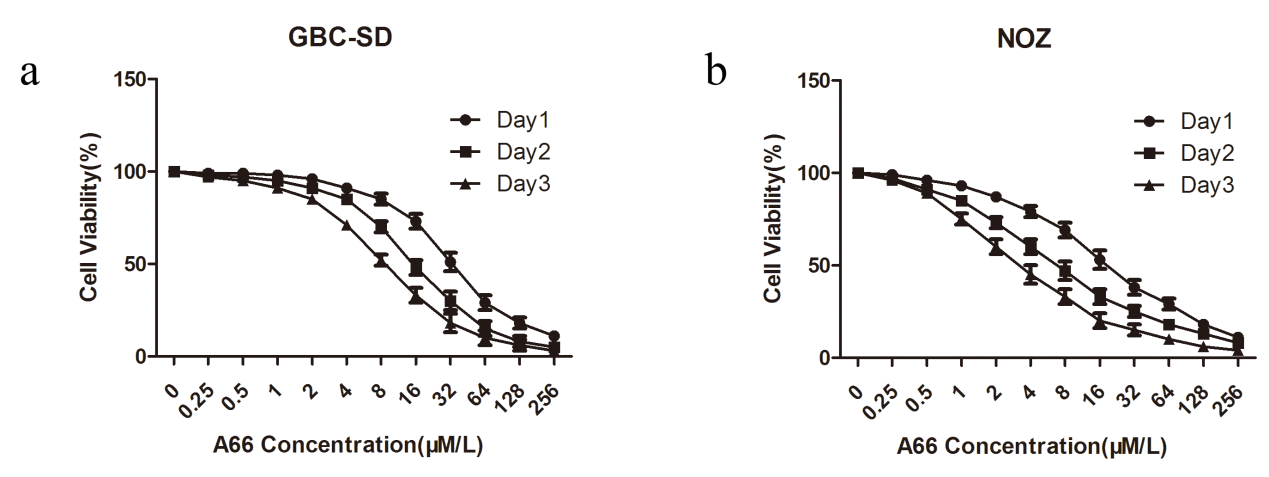


(a, b) GBC-SD and NOZ cells treated with different concentration of A66 were evaluated by CCK8 cell viability assay for 3 days. Data are presented as mean ± SD (n = 5).
